# Evaluation of the Levels of Peripheral CD3^+^, CD4^+^, and CD8^+^ T Cells and IgG and IgM Antibodies in COVID-19 Patients at Different Stages of Infection

**DOI:** 10.1128/spectrum.00845-21

**Published:** 2022-02-23

**Authors:** Waleed Aljabr, Ahod Al-Amari, Basma Abbas, Alaa Karkashan, Saad Alamri, Mohammed Alnamnakani, Athba Al-Qahtani

**Affiliations:** a Research Center, King Fahad Medical City, Riyadh, Kingdom of Saudi Arabia; b Department of Basic Medical Sciences, College of Medicine, Dar Al-Uloom University, Riyadh, Kingdom of Saudi Arabia; c Department of Biological Sciences, College of Science, University of Jeddah, Jeddah, Kingdom of Saudi Arabia; d Pathology and Clinical Laboratory Medicine, King Fahad Medical City, Riyadh, Kingdom of Saudi Arabia; University of Georgia

**Keywords:** SARS-CoV-2, CD4, CD8, T cells, IgG, IgM, COVID-19

## Abstract

Severe acute respiratory syndrome coronavirus 2 (SARS-CoV-2) infection affects the stimulatory levels of cellular-mediated immunity, which plays an essential role in controlling SARS-CoV-2 infection. In fact, several studies have shown the association of lymphopenia with severe COVID-19 in patients. The aim of this study is to investigate the response of the immune system, including cell-mediated immunity and antibody production, during different stages of SARS-CoV-2 infection. Peripheral blood and serum samples were collected from patients with moderate infection, patients under medication (hospitalized), patients who had recovered, and healthy individuals (*n* = 80). Flow cytometry analysis was performed on peripheral blood samples to determine the cellular immunity profile of each patient. The data showed a significant reduction in the levels of CD3^+^, CD4^+^, and CD8^+^ T cells and CD45^+^ cells in the moderate and under-medication groups, suggesting lymphopenia in those patients. Also, enzyme-linked immunosorbent assay (ELISA) was conducted on the serum samples to measure the levels of antibodies, including IgM and IgG, in each patient. The results revealed a significant increase in the levels of IgM in the moderate infection and under-medication patients, thus indicating the production of IgM during the first week of infection. Furthermore, changes in the levels of IgG were significantly detected among recovered patients, indicating therefore a remarkable increase during the recovery stage of SARS-CoV-2 infection and thus a strong humoral-mediated immunity. In summary, the results of this study may help us to understand the main role of the cellular immune responses, including CD3^+^, CD4^+^, and CD8^+^ T cells, against SARS-CoV-2 infection. This understanding might support the development of SARS-CoV-2 treatments and vaccines in the near future.

**IMPORTANCE** Severe acute respiratory syndrome coronavirus 2 (SARS-CoV-2) emerged in late 2019 in China. This virus is a serious threat to people not only in China but also worldwide, where it has been detected in over 222 countries. It has been reported that ∼3.4% of SARS-CoV-2-infected patients have died. The significance of our study relies on the fact that an enzyme-linked immunosorbent assay and flow cytometry were used to measure the levels of antibodies and cellular immune response, respectively, from clinical samples of patients infected with SARS-CoV-2.

## INTRODUCTION

Many years after the emergence of severe acute respiratory syndrome coronavirus (SARS-CoV) in 2003 and the Middle East respiratory syndrome coronavirus (MERS-CoV) in 2012, a new coronavirus spread in China, causing numerous cases of pneumonia in December 2019 (1). The novel coronavirus was named severe acute respiratory syndrome coronavirus 2 (SARS-CoV-2), and the disease was defined as coronavirus infectious disease-19 (COVID-19) (2). Approximately 107 million confirmed cases of COVID-19 and 2 million deaths from it have been reported since December 2019, according to World Health Organization reports (2).

The SARS-CoV-2 disease spectrum varies from asymptomatic infection, to mild disease in the upper respiratory tract or moderate pneumonia, to severe illness with dyspnea and respiratory distress requiring hospitalization and organ support (3, 4). Most reported cases of SARS-CoV-2 are mild to moderate (5, 6). The ratio of patients who require hospitalization increases among individuals above 65 years old and those with underlying medical conditions, such as hypertension, obesity, cardiovascular disease, and diabetes mellitus (5, 6).

The pathogenesis of acute respiratory viral infections and the severity of disease could be modulated either directly by the virus, dysregulated immune responses, or both (7, 8). Several studies have reported that SARS-CoV-2 infection has a greater impact on CD8^+^ T cells than other lymphocytes (7, 9). Other studies have suggested evidence that adaptive immunity, particularly T cells, is critical for preventing severe disease progression in mild and severe COVID-19 cases (5, 9–11). CD4^+^ T cells specific against SARS-CoV-2 spike protein have shown their strong participation in acute infection (12). In other settings, Ki67^+^ CD8^+^ T cells have shown increased numbers in patients with severe COVID-19 (13). Moreover, patients who have recovered from severe SARS-CoV-2 infections have shown strong humoral and cellular immune responses (14). Also, memory CD4^+^ and CD8^+^ T cells have been detected in patients who have recovered from COVID-19 (9, 15). One study has demonstrated that there is an association between the complete restoration of peripheral lymphocytes for patients who have recovered from COVID-19 and viral clearance (16). The duration of immunological memory and whether it can provide protective immunity are still uncertain (9, 15). Although a correlation between the severity of COVID-19 disease and neutralizing antibodies is absent (15), some variants of SARS-CoV-2 have shown their capability to escape the neutralizing humoral immunity that is induced by vaccination (17). In fact, it was suggested that T cell response can mitigate the severity of COVID-19 infection, whereas the presence of neutralizing antibodies is correlated with protective immunity against reinfection with SARS-CoV-2, particularly in nonhuman primates (18, 19).

The reduction of T cells causes lymphopenia, which is very common in various respiratory viral infections (9, 20). Several studies have demonstrated that lymphopenia and inflammatory cytokine storms are associated with severe COVID-19 infections. However, although lymphopenia is not a characteristic feature of COVID-19 infection, the duration of lymphopenia could be more persistent than in other similar coronavirus infections (9, 21–24). The characterization of the immune responses to and immunopathogenicity of COVID-19 infection therefore requires further investigations. Also, variations in neutralizing antibodies and peripheral cell composition, including CD3^+^, CD4^+^, and CD8^+^ cells, should be assessed during different stages of COVID-19 infection. This will gain insight toward understanding the immune responses against SARS-CoV-2.

In this study, peripheral blood samples were collected from COVID-19 patients to compare and identify cellular immune responses, including CD3^+^, CD8^+^, and CD4^+^ T cell production, between different COVID-19 patient groups classified as having moderate infection, hospitalized (under medication), and recovered from COVID-19. Healthy individuals were enrolled as negative controls. IgG and IgM antibodies were measured in the four classified groups.

## RESULTS

### Cell-mediated immunity.

The cellular and humoral responses against SARS-CoV-2 infection were investigated in this study. The response of T cells to COVID-19 infection was analyzed with 80 blood samples from patients with PCR-confirmed COVID-19 in different categories: 20 with moderate infection, 20 under medication, 20 recovered, and 20 healthy. Since both T helper CD4^+^cells and T cytotoxic CD8^+^cells are involved during viral infections, including coronavirus (25, 26), their levels were determined in each patient’s sample.

Flow cytometry analysis was performed on all of the blood cells from all categories except the healthy category, in which only 15 samples were included, to generate the profile and levels of immune cells subsets in each sample. The level of immune cells was evaluated by calculating the median percentage of cells obtained from each sample. Then, a correlation between the level of immune cells and different groups’ status (categories) was determined. The results revealed different levels of CD3^+^, CD4^+^, CD8^+^, CD16^+^ CD56^+^, CD19^+^, and CD45^+^ between the groups. Also, the results showed different ratios of CD4^+^ to CD8^+^ T cells: this ratio compares the portion of helper CD4^+^ T cells to killer CD8^+^ T cells.

**FIG 1:**
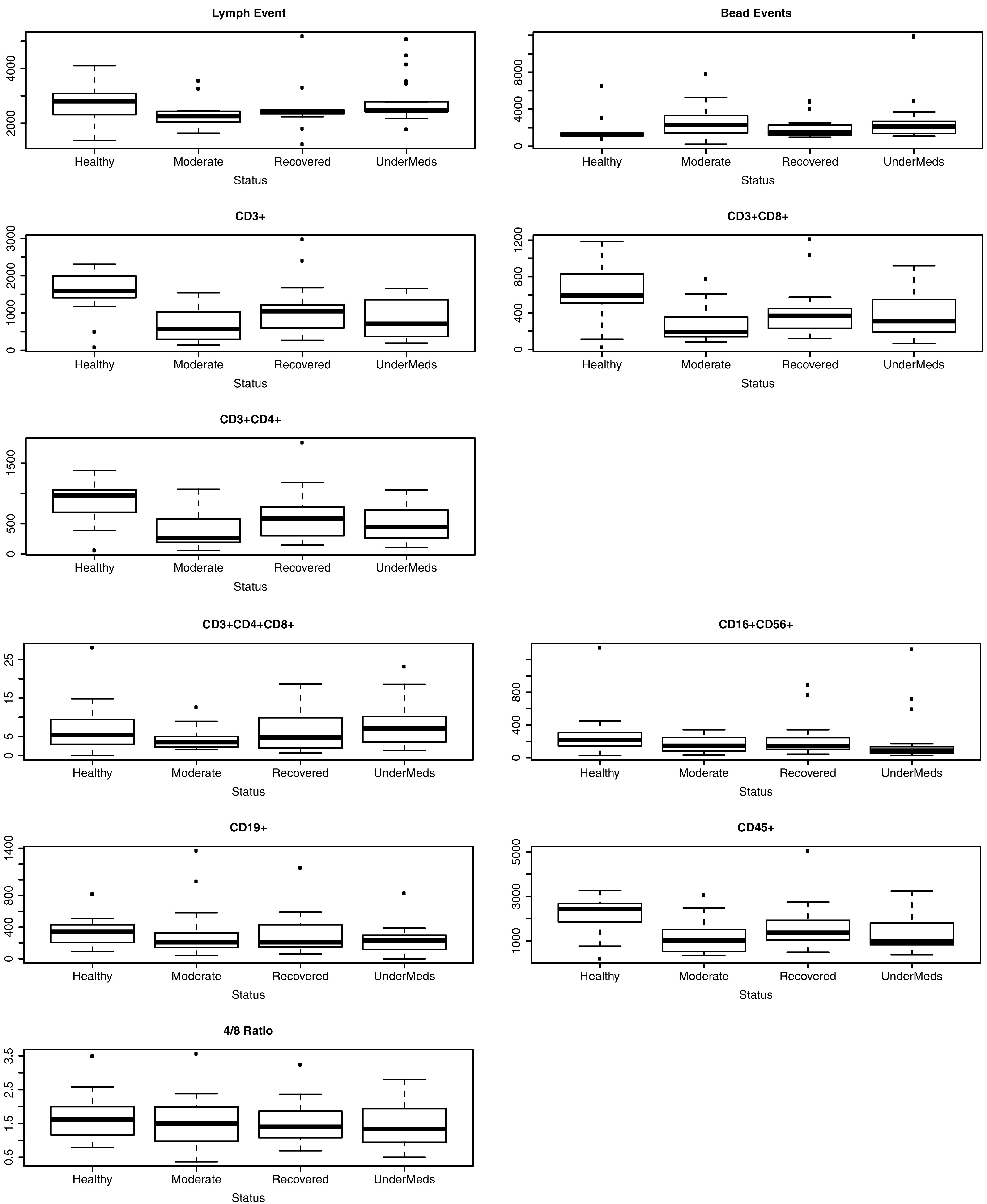
Box plot showing the level of immune cells (lymph events, bead events, CD3^+^, CD3^+^ CD8^+^, CD3^+^ CD4^+^, CD3^+^ CD4^+^ CD8^+^, CD16^+^ CD56^+^, CD19^+^, CD45^+^, and CD4^+^/CD8^+^ T cell ratio) of the study group was evaluated using flow cytometry. The box plot shows the data distribution and shows the outliers. The line in the middle represents the median of the percentage of cells obtained from each sample. Results were considered significant at *P < *0.05.

As shown in Table 1 and Fig. 1, significant differences in the levels of CD3^+^, CD4^+^, CD8^+^, and CD45^+^ cells between the study groups (*P* < 0.05 by analysis of variance [ANOVA]) were observed. The cells were significantly higher in the healthy group compared to the moderate infection and under-medication individuals. However, there was an insignificant difference in the levels of CD3^+^ cells between the healthy and recovered patients (*P* < 0.05 by Tukey’s honestly significant difference [HSD] *post hoc* test) (Table 2 and Fig. 1).

**TABLE 1:** ANOVA results

ANOVA parameter	Sum of squares	df	Mean square	*F*	Significance
Lymph events					
Between groups	2,421,353.063	3	807,117.688	1.503	0.222
Within groups	3.653E7	68	537,164.062		
Total	3.895E7	71			

Bead events					
Between groups	2.371E7	3	7,904,048.484	1.771	0.161
Within groups	3.034E8	68	4,461,942.383		
Total	3.271E8	71			

CD3^+^					
Between groups	6,666,720.701	3	2,222,240.234	6.575	0.001
Within groups	2.298E7	68	337,975.802		
Total	2.965E7	71			

CD3^+^ CD8^+^					
Between groups	1,060,627.022	3	353,542.341	5.248	0.003
Within groups	4,580,919.774	68	67,366.467		
Total	5,641,546.796	71			

CD3^+^ CD4^+^					
Between groups	2,025,826.300	3	675,275.433	5.271	0.003
Within groups	8,711,165.749	68	128,105.379		
Total	1.074E7	71			

CD3^+^ CD4^+^ CD8^+^					
Between groups	127.978	3	42.659	1.484	0.227
Within groups	1,954.833	68	28.748		
Total	2,082.812	71			

CD16^+^ CD56^+^					
Between groups	139,806.392	3	46,602.131	0.716	0.546
Within groups	4,424,094.281	68	65,060.210		
Total	4,563,900.673	71			

CD19^+^					
Between groups	109,535.879	3	36,511.960	0.583	0.628
Within groups	4,260,738.831	68	62,657.924		
Total	4,370,274.711	71			

CD45^+^					
Between groups	9,692,126.568	3	3,230,708.856	4.423	0.007
Within groups	4.967E7	68	730,438.296		
Total	5.936E7	71			

CD4^+^/CD8^+^ ratio					
Between groups	0.280	3	0.093	0.178	0.911
Within groups	35.677	68	0.525		
Total	35.957	71			

**TABLE 2:** Tukey’s HSD *post hoc* test for multiple comparisons

Dependent variable	Patient status^*a*^		Mean difference (I − J)^*b*^	SE	Significance	95% CI^*c*^	
						Lower bound	Upper bound
	I	J					
Lymph events	Healthy	Moderate	384.161	259.632	0.455	−299.64	1,067.96
		Recovered	244.867	253.146	0.768	−421.85	911.58
		Under meds	−67.752	247.770	0.993	−720.31	584.80
	Moderate	Healthy	−384.161	259.632	0.455	−1,067.96	299.64
		Recovered	−139.294	244.683	0.941	−783.72	505.13
		Under meds	−451.913	239.117	0.242	−1,081.68	177.85
	Recovered	Healthy	−244.867	253.146	0.768	−911.58	421.85
		Moderate	139.294	244.683	0.941	−505.13	783.72
		Under meds	−312.619	232.058	0.537	−923.79	298.56
	Under meds	Healthy	67.752	247.770	0.993	−584.80	720.31
		Moderate	451.913	239.117	0.242	−177.85	1,081.68
		Recovered	312.619	232.058	0.537	−298.56	923.79

Bead events	Healthy	Moderate	−1,058.447	748.285	0.495	−3,029.22	912.32
		Recovered	−330.958	729.590	0.969	−2,252.49	1,590.58
		Under meds	−1,451.324	714.098	0.186	−3,332.06	429.41
	Moderate	Healthy	1,058.447	748.285	0.495	−912.32	3,029.22
		Recovered	727.489	705.199	0.732	−1,129.81	2,584.79
		Under meds	−392.877	689.159	0.941	−2,207.93	1,422.17
	Recovered	Healthy	330.958	729.590	0.969	−1,590.58	2,252.49
		Moderate	−727.489	705.199	0.732	−2,584.79	1,129.81
		Under meds	−1,120.366	668.814	0.345	−2,881.83	641.10
	Under meds	Healthy	1,451.324	714.098	0.186	−429.41	3,332.06
		Moderate	392.877	689.159	0.941	−1,422.17	2,207.93
		Recovered	1,120.366	668.814	0.345	−641.10	2,881.83

CD3^+^	Healthy	Moderate	868.04561*	2.05943E2	0.000	325.6492	1,410.4420
		Recovered	473.35372	2.00798E2	0.095	−55.4920	1,002.1994
		Under meds	676.41171*	1.96534E2	0.005	158.7955	1,194.0280
	Moderate	Healthy	−868.04561*	2.05943E2	0.000	−1,410.4420	−325.6492
		Recovered	−394.69189	1.94085E2	0.186	−905.8578	116.4741
		Under meds	−191.63389	1.89671E2	0.744	−691.1731	307.9054
	Recovered	Healthy	−473.35372	2.00798E2	0.095	−1,002.1994	55.4920
		Moderate	394.69189	1.94085E2	0.186	−116.4741	905.8578
		Under meds	203.05799	1.84071E2	0.689	−281.7340	687.8500
	Under meds	Healthy	−676.41171*	1.96534E2	0.005	−1,194.0280	−158.7955
		Moderate	191.63389	1.89671E2	0.744	−307.9054	691.1731
		Recovered	−203.05799	1.84071E2	0.689	−687.8500	281.7340

CD3^+^ CD8^+^	Healthy	Moderate	353.59616*	91.94472	0.001	111.4398	595.7525
		Recovered	213.99839	89.64765	0.089	−22.1082	450.1049
		Under meds	259.02400*	87.74409	0.022	27.9309	490.1171
	Moderate	Healthy	−353.59616*	91.94472	0.001	−595.7525	−111.4398
		Recovered	−139.59777	86.65066	0.379	−367.8111	88.6156
		Under meds	−94.57216	84.67975	0.680	−317.5947	128.4504
	Recovered	Healthy	−213.99839	89.64765	0.089	−450.1049	22.1082
		Moderate	139.59777	86.65066	0.379	−88.6156	367.8111
		Under meds	45.02561	82.17987	0.947	−171.4129	261.4641
	Under meds	Healthy	−259.02400*	87.74409	0.022	−490.1171	−27.9309
		Moderate	94.57216	84.67975	0.680	−128.4504	317.5947
		Recovered	−45.02561	82.17987	0.947	−261.4641	171.4129

CD3^+^ CD4^+^	Healthy	Moderate	470.64486*	1.26791E2	0.002	136.7131	804.5766
		Recovered	231.44186	1.23623E2	0.250	−94.1472	557.0309
		Under meds	374.43990*	1.20998E2	0.015	55.7643	693.1155
	Moderate	Healthy	−470.64486*	1.26791E2	0.002	−804.5766	−136.7131
		Recovered	−239.20300	1.19491E2	0.197	−553.9074	75.5014
		Under meds	−96.20496	1.16773E2	0.843	−403.7513	211.3413
	Recovered	Healthy	−231.44186	1.23623E2	0.250	−557.0309	94.1472
		Moderate	239.20300	1.19491E2	0.197	−75.5014	553.9074
		Under meds	142.99805	1.13325E2	0.590	−155.4690	441.4651
	Under meds	Healthy	−374.43990*	1.20998E2	0.015	−693.1155	−55.7643
		Moderate	96.20496	1.16773E2	0.843	−211.3413	403.7513
		Recovered	−142.99805	1.13325E2	0.590	−441.4651	155.4690

CD3^+^ CD4^+^ CD8^+^	Healthy	Moderate	3.05867	1.89935	0.380	−1.9437	8.0610
		Recovered	1.27025	1.85190	0.902	−3.6071	6.1476
		Under meds	−0.37562	1.81258	0.997	−5.1494	4.3982
	Moderate	Healthy	−3.05867	1.89935	0.380	−8.0610	1.9437
		Recovered	−1.78842	1.78999	0.750	−6.5027	2.9259
		Under meds	−3.43429	1.74927	0.212	−8.0414	1.1728
	Recovered	Healthy	−1.27025	1.85190	0.902	−6.1476	3.6071
		Moderate	1.78842	1.78999	0.750	−2.9259	6.5027
		Under meds	−1.64586	1.69763	0.767	−6.1170	2.8252
	Under meds	Healthy	0.37562	1.81258	0.997	−4.3982	5.1494
		Moderate	3.43429	1.74927	0.212	−1.1728	8.0414
		Recovered	1.64586	1.69763	0.767	−2.8252	6.1170

CD16^+^ CD56^+^	Healthy	Moderate	126.61435	90.35717	0.503	−111.3609	364.5896
		Recovered	58.26147	88.09977	0.911	−173.7684	290.2913
		Under meds	91.21676	86.22907	0.716	−135.8862	318.3197
	Moderate	Healthy	−126.61435	90.35717	0.503	−364.5896	111.3609
		Recovered	−68.35288	85.15452	0.853	−292.6258	155.9200
		Under meds	−35.39759	83.21764	0.974	−254.5693	183.7741
	Recovered	Healthy	−58.26147	88.09977	0.911	−290.2913	173.7684
		Moderate	68.35288	85.15452	0.853	−155.9200	292.6258
		Under meds	32.95529	80.76092	0.977	−179.7461	245.6567
	Under meds	Healthy	−91.21676	86.22907	0.716	−318.3197	135.8862
		Moderate	35.39759	83.21764	0.974	−183.7741	254.5693
		Recovered	−32.95529	80.76092	0.977	−245.6567	179.7461

CD19^+^	Healthy	Moderate	9.14118	88.67331	1.000	−224.3992	242.6816
		Recovered	23.15000	86.45797	0.993	−204.5558	250.8558
		Under meds	95.64048	84.62213	0.672	−127.2303	318.5112
	Moderate	Healthy	−9.14118	88.67331	1.000	−242.6816	224.3992
		Recovered	14.00882	83.56761	0.998	−206.0846	234.1023
		Under meds	86.49930	81.66683	0.715	−128.5880	301.5866
	Recovered	Healthy	−23.15000	86.45797	0.993	−250.8558	204.5558
		Moderate	−14.00882	83.56761	0.998	−234.1023	206.0846
		Under meds	72.49048	79.25589	0.797	−136.2471	281.2281
	Under meds	Healthy	−95.64048	84.62213	0.672	−318.5112	127.2303
		Moderate	−86.49930	81.66683	0.715	−301.5866	128.5880
		Recovered	−72.49048	79.25589	0.797	−281.2281	136.2471

CD45^+^	Healthy	Moderate	1,009.16992*	3.02759E2	0.007	211.7891	1,806.5508
		Recovered	560.00302	2.95195E2	0.239	−217.4567	1,337.4628
		Under meds	869.87267*	2.88927E2	0.019	108.9214	1,630.8240
	Moderate	Healthy	−1,009.16992*	3.02759E2	0.007	−1,806.5508	−211.7891
		Recovered	−449.16690	2.85326E2	0.400	−1,200.6356	302.3018
		Under meds	−139.29725	2.78836E2	0.959	−873.6734	595.0789
	Recovered	Healthy	−560.00302	2.95195E2	0.239	−1,337.4628	217.4567
		Moderate	449.16690	2.85326E2	0.400	−302.3018	1,200.6356
		Under meds	309.86965	2.70605E2	0.663	−402.8265	1,022.5658
	Under meds	Healthy	−869.87267*	2.88927E2	0.019	−1,630.8240	−108.9214
		Moderate	139.29725	2.78836E2	0.959	−595.0789	873.6734
		Recovered	−309.86965	2.70605E2	0.663	−1,022.5658	402.8265

CD4^+^/CD8^+^ ratio	Healthy	Moderate	0.11329	0.25659	0.971	−0.5625	0.7891
		Recovered	0.12905	0.25018	0.955	−0.5299	0.7880
		Under meds	0.17610	0.24487	0.889	−0.4688	0.8210
	Moderate	Healthy	−0.11329	0.25659	0.971	−0.7891	0.5625
		Recovered	0.01576	0.24182	1.000	−0.6211	0.6526
		Under meds	0.06280	0.23632	0.993	−0.5596	0.6852
	Recovered	Healthy	−0.12905	0.25018	0.955	−0.7880	0.5299
		Moderate	−0.01576	0.24182	1.000	−0.6526	0.6211
		Under meds	0.04704	0.22934	0.997	−0.5570	0.6511
	Under meds	Healthy	−0.17610	0.24487	0.889	−0.8210	0.4688
		Moderate	−0.06280	0.23632	0.993	−0.6852	0.5596
		Recovered	−0.04704	0.22934	0.997	−0.6511	0.5570

a Moderate, patients with moderate infection; Under meds, patients under medication (hospitalized).

b *, the mean difference is significant at the 0.05 level.

c 95% CI, 95% confidence interval.

Regarding the other components of the immune profile, including CD16^+^, CD56^+^, CD19^+^, and CD4^+^/CD8^+^ T cell ratio, the data show an insignificant difference between the study groups, as shown in Tables 1 and 2 and Fig. 1 (*P* < 0.05 by ANOVA) (*P* < 0.05 by Tukey’s HSD *post hoc* test).

Furthermore, a correlation matrix plot was used to assess the level of correlation between different immune cells. A strong correlation was found between CD4^+^ and CD45^+^ cells, CD3^+^ and CD4^+^ cells, CD8^+^ cells, and CD45^+^ cells. Correlations were also found with CD8^+^ and CD4^+^ cells and CD45^+^ cells. In addition, a correlation was observed between CD19^+^ and CD45^+^ cells, as shown in Fig. S1 in the supplemental material. The results revealed that CD3^+^, CD4^+^, CD8^+^, CD45^+^, and CD19^+^ cells had a strong relationship and high level of correlation with each other in a specific manner as a response to SARS-CoV-2 infection.

### Antibody production.

In addition to the T cell response, production of IgG and IgM antibodies against SARS-CoV-2 infection were investigated in this study by enzyme-linked immunosorbent assay (ELISA). ELISA is a serological test used to detect the presence of the immunoglobulins IgG and IgM against SARS-CoV-2 (27, 28), targeting the spike and nucleocapsid proteins (S and N, respectively). Four categories were involved and included the following numbers of patients: 20 who were healthy, 20 with moderate infection, 20 under medication, and 20 who had recovered.

**FIG 2:**
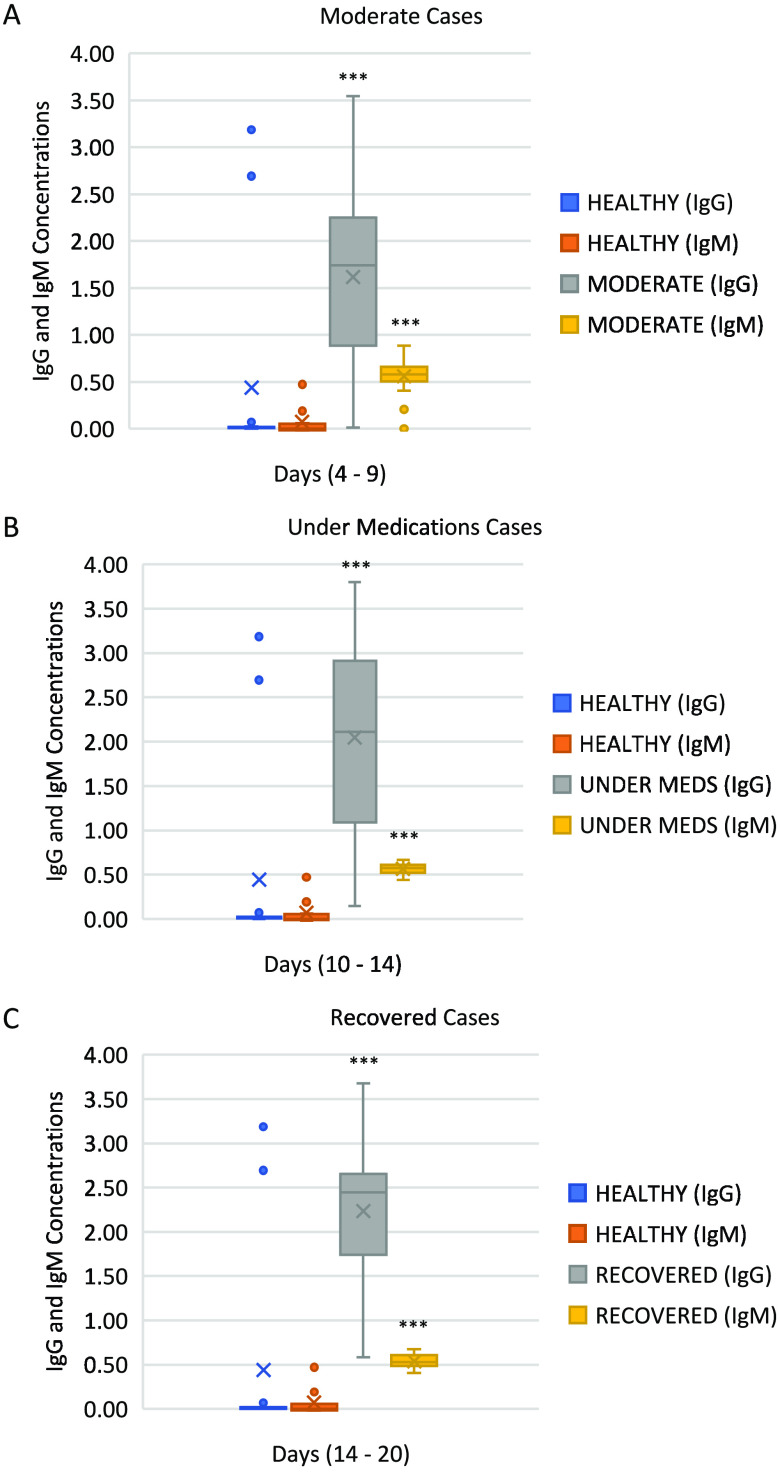
Illustration of IgG and IgM levels in different groups of patients. The levels of IgG and IgM were determined using ELISA in three different groups: (A) moderate group, (B) under-medication group, and (C) recovered group. Results were considered significant at *P < *0.001 (***).

As seen in Fig. 2, the levels of IgM in the moderate infection, under-medication, and recovered groups were significantly higher than those in the healthy group (*P* < 0.001 by ANOVA). Although IgM levels showed an insignificant difference between the moderate infection and under-medication groups, both had significantly higher levels than the recovered individuals (*P* < 0.001 by ANOVA). IgG, on the other hand, has shown similar results to IgM in terms of their presence across the categories. IgG levels were significantly lower in the healthy group compared to the other groups, including the individuals in the moderate infection, under-medication, and recovered groups (Fig. 2) (*P* < 0.001 by ANOVA). Although IgG levels in the moderate infection and under-medication groups were significantly higher than those in the healthy group, they were significantly lower than those in the recovered individuals (*P* < 0.001 by ANOVA). The levels of IgG in the moderate infection group showed a significant decrease compared to the under-medication group as well as the recovered group. The IgG levels in the under-medication group were significantly higher than in the moderate infection group but significantly lower than those in the recovered group (*P* < 0.001 by ANOVA). Finally, patients who have recovered from COVID-19 showed the highest level of IgG in comparison to the other groups (*P* < 0.001 by ANOVA). Interestingly, the levels of IgG antibody in the recovered, moderate infection, and under-medication groups were higher than IgM antibody levels in the same groups.

**FIG 3:**
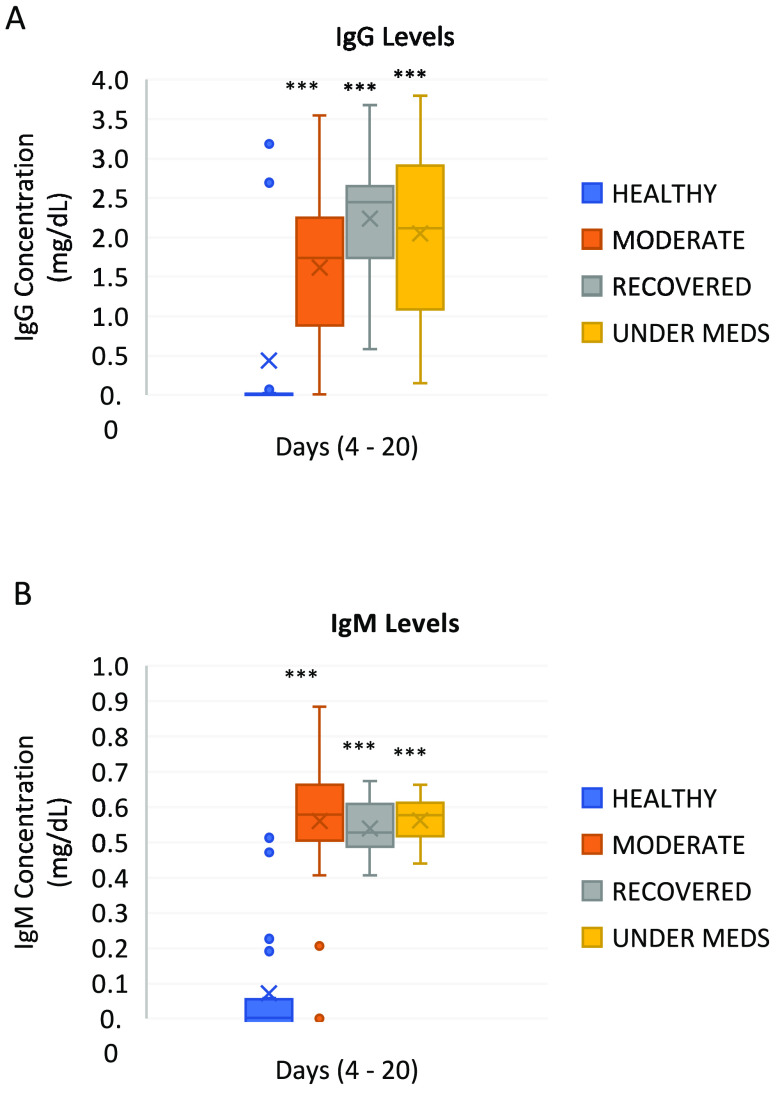
Levels of IgG and IgM in healthy, moderate, under-medication, and recovered patients. The levels of IgG and IgM immunoglobulins were determined using commercially available ELISAs to determine concentrations of (A) IgG and (B) IgM. Results were considered significant at *P < *0.001 (***).

Moreover, the levels of IgG increased as the recovery state developed from healthy, to moderate infection, to under medications, to recovered. The recovered group exhibited the highest levels of IgG immunity after infection among different groups, thus indicating an increase in the levels of memory immunity, as illustrated in Fig. 3. The levels of IgM, on the other hand, decreased as recovery advanced. The levels of IgM were higher in the moderate infection group and decreased as the patients were recovering (Fig. 3).

In summary, we could determine the role of CD3^+^, CD4^+^, and CD8^+^ T cells, as well as CD45^+^ cells, as they showed impaired cellular immunity in infected patients. The results for IgM and IgG antibodies indicated, on the other hand, a strong humoral immune response in SARS-CoV-2 patients.

## DISCUSSION

Cellular immune responses play an essential role in virus clearance and disease severity (29–31). One study on SARS-CoV has demonstrated the requirement of specific T cell responses for the treatment of SARS-CoV-infected mice (29). In addition, several studies have shown that cell-mediated immunity, mainly cytotoxic (CD8^+^), is an essential factor in eliminating numerous virus-infected cells during severe infections (reviewed in reference 26). On the other hand, a study has demonstrated that depletion of helper T cells (CD4^+^) in SARS-Cov-infected mice increases the duration of viral clearance, whereas there was no effect when CD8^+^ T cells were depleted (30). This indicates the important role of CD4^+^ T cells in controlling SARS-Cov infection, as well as other viral infections (30, 32). Specific CD4^+^ T cells were detected in peripheral blood samples of COVID-19 patients (33). Several studies have investigated the T cell responses of COVID-19 patients; however, most of them were performed on a single patient or small number of patients (7). Besides, information regarding the correspondence between levels of T cell responses (CD4^+^ and CD8^+^) during different COVID-19 stages is scarce—hence the importance of this study. This study was performed using peripheral blood samples from 80 individuals with different stages of COVID-19. Flow cytometry was used to detect the populations of CD4^+^ CD8^+^ and CD3^+^ T cells. Also, specific IgM and IgG antibodies were detected using ELISA. Differences were observed with population levels of CD4^+^ CD8^+^ and CD3^+^ T cells at different stages of SARS-CoV-2 infection.

The results of the current study indicated a significant reduction in the levels of CD3^+^, CD4^+^, and CD8^+^ T cells in the moderate infection and under-medication groups compared to the healthy group. This finding is in agreement with what was observed by previous studies, as the results showed a low count of CD3^+^, CD4^+^, and CD8^+^ T cells in COVID-19 patients, particularly in moderate and severe cases (20, 34, 35). However, the recovered COVID-19 patients in this study showed an increase in CD3^+^, CD8^+^, and CD4^+^ T cell levels in comparison to the moderate infection and under-medication groups. Therefore, it can be deduced that SARS-CoV-2 infection could affect T cell population counts in the peripheral blood, most frequently with severe cases (11, 34). When the T cell levels were compared between the group that recovered from COVID-19 and healthy controls, they showed an insignificant difference. Restoration of T cells in the recovered patients could probably be an indication of viral clearance (16). Moreover, the CD45^+^ level was decreased significantly in the moderate infection and under-medication groups in the present study. This is similar to what was observed in previous studies that showed a decline in the level of CD45^+^ in severe COVID-19 patients (34, 36). CD45^+^ is an important transmembrane phosphatase involved in T cell activation and signaling (36). It has been demonstrated that the expression of CD45^+^ on T cells was decreased in HIV infection (36). This suggests that CD45^+^ could be used also as an indicator for assessment of the severity of COVID-19 disease (36).

The reduction in T cells, mainly CD4^+^ and CD8^+^, is common between severe COVID-19 cases (9). This attribute was also observed in the present study with the moderate infection and under-medication groups, suggesting that there is a relationship between COVID-19 disease severity and the occurrence of lymphopenia (34). Although lymphopenia is a common feature of many respiratory viral infections, such as human rhinovirus, SARS-CoV, and MERS-CoV, it is more persistent with SARS-CoV-2, likely due to selection of those T cell lineages rather than the other ones. This is by affecting CD4^+^ T cells, CD8^+^ T cells, B cells, and natural killer cells (9, 20), a phenomenon found in this study as well. Detection of lymphopenia in COVID-19 patients might thus affect the cell-mediated response, which has a principal role in eliminating viral infection. In addition, lymphopenia affects other immune cells, including B cells, innate immune cells, and natural killer cells. In fact, there was an insignificant difference in the ratio of CD4^+^ to CD8^+^ T cells between the groups, although CD4^+^ and CD8^+^ T cells play a vital role in maintaining immune function and viral clearance in the body. This finding was also observed in another study that showed an insignificant difference in the CD4^+^/CD8^+^ T cell ratio between COVID-19 patients and healthy individuals (25).

The immunoglobulins IgG and IgM are usually generated by the adaptive immune system in response to infections (37). IgG and IgM can be specific and serve as diagnostic markers for detecting infections such as COVID-19 (38). The results of this study showed that the levels of IgG and IgM antibodies are increased in SARS-CoV-2 patients (Fig. 3). This finding is identical to the results of another group that showed a positive IgG titer in patients infected with SARS-CoV-2 during the first 3 weeks after symptom onset, although IgM showed a slight decrease in the third week (39). The latter was also observed in our study, as IgM levels started to be generated in the moderate infection and under-mediation groups, and then the levels began to decline in the recovered individuals, suggesting that IgM could be produced first in response to the SARS-CoV-2 infection at the early stage since it can be expressed without isotype switching. Moreover, other studies have reported that IgM levels increased in patients in the first week of infection, with a high peak in the second week prior to a great reduction near background levels in most patients. IgG antibody, on the other hand, was observed after 1 week of infection and was maintained at a high level for a long period of time after the infection occurred (40, 41). The latter results are similar to what was observed in this study, as IgG levels showed an increase in the moderate stage of infection, reaching the highest level in the recovery stage. High IgG levels were maintained until day 20. This indicates that the patients have developed a humoral immune response against COVID-19 within 7 to 10 days after they have had the infection. Moreover, these results suggest that the IgM antibodies were produced as the body is still infected; however, when the body starts to recover, the IgG antibody starts to be produced, as reported earlier (42). The production of IgM and IgG antibodies during infection and the increase in IgG in the recovery stage suggest also that IgM and IgG may have neutralized the SARS-CoV-2 and that IgG antibodies created immunity for the body to fight current and future infections.

Another study has investigated IgG and IgM responses to SARS-CoV-2 nucleocapsid (N) and spike (S) proteins in severe and nonsevere cases after infection (43). The study reported that the response of IgM against S and N proteins increases after the onset of symptoms, reaching a peak in the second week of infection in some patients. The response of IgG against N and S proteins, however, continued to increase in the third week in some patients. The response of IgG to S protein was higher in moderate cases in comparison to severe cases in the third week. The response of IgG against N protein, on the other hand, was significantly higher in severe cases, with this difference attributed to the fact that the increase in IgG in response to S protein correlated with a reduction in C-reactive proteins in severely infected patients (43). In our study, we have noticed a similar pattern where levels of IgG increased considerably. Therefore, it would be interesting to investigate the levels of IgG and IgM in response to N proteins as we have only measured the levels of IgG and IgM against the S protein in this set of patients. Also, quantitative detection of IgG and IgM could help in evaluating the severity of the disease as well as could establish a dynamic that can help in predicting prognosis.

In conclusion, our data present the responses of cell-mediated and humoral-mediated immunity in both infected and recovered SARS-CoV-2 patients. The results support the concept of lymphopenia in patients with moderate infection and under medication, as confirmed in other studies (9, 20). On the other hand, our study confirmed the presence of strong humoral-mediated immunity in the recovered cases. It could be speculated that SARS-CoV-2 infection negatively affected the activation and proliferation of T cells, while the production of antibodies occurred independently of T cells. The latter represents the role of humoral immunity in controlling SARS-CoV-2 infection and in providing the required protection, which may be investigated in future studies for their long-term presence. Furthermore, our results can aid in the future development of SARS-CoV-2 treatments and vaccines. In addition, our results can enhance the discovery of anti-virus-specific T cell clones and the development new immunotherapeutic “weapons” to fight this threatening disease.

## MATERIALS AND METHODS

### Ethical considerations.

A total of 80 samples (*n* = 80) were collected with informed consent under ethical approval from the Institutional Review Board (no. 20-162) of King Fahad Medical City, Riyadh, Kingdom of Saudi Arabia (KSA).

### Sample collection and processing.

Eighty samples were collected from patients (49 male and 31 female, complete age range of 21 to 91 years, and average age of 51 years) at the Prince Mohammed Bin Abdulaziz Hospital (PMAH), Riyadh, KSA. None of the included patients had received a SARS-CoV-2 vaccine. The SARS-CoV-2 diagnosis was confirmed by reverse transcriptase PCR (RT-PCR) (bioMérieux Diagnostics). The cohort of the study included healthy individuals (>15 years old) with no history of respiratory illnesses, smoking, or obesity, patients with a moderate stage of infection (>15 years old, with samples collected between 4 and 9 days [average of 6.5 days] from the day of reporting COVID-19 positive), patients under medication who received medical treatments upon admission (hospitalized) (>15 years old, with samples collected between 10 and 14 days [average of 12 days] from the day of reporting COVID-19 positive), and recovered patients (>15 years old, with samples collected between 14 and 20 days [average of 17 days] from the day of reporting COVID-19 positive).

Blood samples (two tubes from each patient) from patients with confirmed SARS-CoV-2 diagnosis as well as from SARS-CoV-2-negative patients (who had no symptoms of SARS-CoV-2 infection and no identifying information) from the PMAH were utilized in this study. The first fresh blood tube was directly utilized in the flow cytometry experiment. For the ELISA, the serum from the second blood tube was isolated and stored at −80°C until use.

### Flow cytometry.

Each patient’s sample was placed in a BD Trucount tube that was labeled with the sample accession number. Ten microliters of BD Multitest 6-color TBNK reagent was added, followed by 50 μL of a well-mixed EDTA-anticoagulated whole-blood sample. The tube was then capped and vortexed gently to be mixed. The sample was incubated for 15 min in the dark at room temperature (20 to 25°C). Thereafter, 450 μL of 1× BD fluorescence-activated cell sorter (FACS) lysing solution was added. The sample was capped and vortexed gently and then incubated for 10 min in the dark at room temperature (20 to 25°C). The sample should have been acquired within 1 h of lysing. The sample of viable total cells was ready to be analyzed on the flow cytometer. In each sample, the levels that represent the percentages of total cell numbers of CD3^+^/CD3^+^ CD4^+^/CD3^+^ CD8^+^ T cells, CD19^+^ B lymphocytes, and CD45^+^ cells were calculated for each patient. Analysis was performed using the BD FACSCanto clinical software.

### ELISA.

In order to detect the presence of the spike and nucleocapsid (S and N) proteins of the SARS-CoV-2 virus, commercially available SARS-CoV-2 IgG antibody and SARS-CoV-2 IgM antibody detection kits (from BGI, catalogue no. 0601038 and 0601039, respectively) were used as per the manufacturer’s instructions. All reagents were incubated at room temperature for half an hour prior to use. Washing buffer was diluted at a ratio of 1:20 with distilled water. Serum samples were diluted at a ratio of 1:10 in reagent diluent. One hundred microliters of diluted samples was added to each well, along with 100 μL of each positive and negative control. A blank well where no liquid was added was included. The coated plate was incubated at 37°C for 60 min. The plate was then washed five times using 300 μL washing buffer with the assistance of an automated microplate washer (Wellwash; Thermo Fisher). One hundred microliters of the enzyme solution was added to each well, and the plate was incubated at 37°C for 20 min. Thereafter, the plate was washed with washing buffer five times. Then 50 μL of both substrates A and B was added to each well and mixed thoroughly. The plate was incubated at 37°C for 10 min away from light prior to quenching the reaction using 50 μL of stop buffer added to each well. The absorbance was measured immediately at a 450-nm wavelength using a Multiskan microplate reader purchased from Thermo Scientific.

### Statistical analysis.

Statistical analysis was done using RStudio version 1.4.1103 and IBM SPSS 16.0. Correlation and data exploration were performed to understand the variables. ANOVA, which is a collection of methods for comparing multiple means across different groups, was done for all four categories across variables. A *post hoc* comparison HSD Tukey’s test was performed to find the differences between the categories. A two-sided *P* value of <0.05 in flow cytometry data and a *P* value of <0.001 in ELISA data were considered statistically significant with a 95% confidence level.

## References

[1] Oliveira DS, Medeiros NI, Gomes JAS. 2020. Immune response in COVID-19: what do we currently know? Microb Pathog 148:104484. doi:10.1016/j.micpath.2020.104484. 32916246 PMC7480770

[2] World Health Organization. 2020. Origin of SARS-CoV-2. https://www.who.int/publications/i/item/origin-of-sars-cov-2.

[3] Carsetti R, Zaffina S, Mortari EP, Terreri S, Corrente F, Capponi C, Palomba P, Mirabella M, Cascioli S, Palange P, Cuccaro I, Milito C, Zumla A, Maeurer M, Camisa V, Vinci MR, Santoro A, Cimini E, Marchioni L, Nicastri E, Palmieri F, Agrsti C, Ippolito G, Porzio O, Concato C, Muda AO, Raponi M, Quintarelli C, Quinti I, Locatelli F. 2020. Different innate and adaptive immune response to SARS-CoV-2 infection of asymptomatic, mild and severe cases. medRxiv. https://www.medrxiv.org/content/medrxiv/early/2020/09/28/2020.06.22.20137141.full.pdf. 10.3389/fimmu.2020.610300 PMC7772470 33391280

[4] Singhal T. 2020. A review of coronavirus disease-2019 (COVID-19). Indian J Pediatr 87:281–286. doi:10.1007/s12098-020-03263-6. 32166607 PMC7090728

[5] Rydyznski Moderbacher C, Ramirez SI, Dan JM, Grifoni A, Hastie KM, Weiskopf D, Belanger S, Abbott RK, Kim C, Choi J, Kato Y, Crotty EG, Kim C, Rawlings SA, Mateus J, Tse LPV, Frazier A, Baric R, Peters B, Greenbaum J, Ollmann Saphire E, Smith DM, Sette A, Crotty S. 2020. Antigen-specific adaptive immunity to SARS-CoV-2 in acute COVID-19 and associations with age and disease severity. Cell 183:996–1012.e19. doi:10.1016/j.cell.2020.09.038. 33010815 PMC7494270

[6] Garg S, Kim L, Whitaker M, Cummings C, Holstein R, Prill M, Chai SJ, Kirley PD, Alden NB, Kawasaki B, Yousey-Hindes K, Niccolai L, Anderson EJ, Openo KP, Weigel A, Monroe ML, Ryan P, Henderson J, Kim S, Como-Sabetti K, Lynfield R, Sosin D, Torres S, Muse A, Bennett NM, Billing L, Sutton M, West N, Schaffner W, Keipp Talbot H, Aquino C, George A, Budd A, Brammer L, Langley G, Hall AJ, Fry A. 2019. Hospitalization rates and characteristics of patients hospitalized with laboratory-confirmed coronavirus disease 2019—COVID-NET, 14 states, March 1–30, 2020. MMWR Morb Mortal Wkly Rep 69:458–464. doi:10.15585/mmwr.mm6915e3. PMC7755063 32298251

[7] Mathew D, Giles JR, Baxter AE, Oldridge DA, Greenplate AR, Wu JE, Alanio C, Kuri-Cervantes L, Pampena MB, D’Andrea K, Manne S, Chen Z, Huang YJ, Reilly JP, Weisman AR, Ittner CAG, Kuthuru O, Dougherty J, Nzingha K, Han N, Kim J, Pattekar A, Goodwin EC, Anderson EM, Weirick ME, Gouma S, Arevalo CP, Bolton MJ, Chen F, Lacey SF, Ramage H, Cherry S, Hensley SE, Apostolidis SA, Huang AC, Vella LA, Betts MR, Meyer NJ, Wherry EJ, Alam Z, Addison MM, Byrne KT, Chandra A, Descamps HC, Kaminskiy Y, Hamilton JT, Noll JH, Omran DK, Perkey E, Prager EM, et al. 2020. Deep immune profiling of COVID-19 patients reveals distinct immunotypes with therapeutic implications. Science 369:eabc8511. doi:10.1126/science.abc8511. 32669297 PMC7402624

[8] Tay MZ, Poh CM, Rénia L, MacAry PA, Ng LFP. 2020. The trinity of COVID-19: immunity, inflammation and intervention. Nat Rev Immunol 20:363–374. doi:10.1038/s41577-020-0311-8. 32346093 PMC7187672

[9] Chen Z, Wherry JE. 2020. T cell responses in patients with COVID-19. Nat Rev Immunol 20:529–536. doi:10.1038/s41577-020-0402-6. 32728222 PMC7389156

[10] Diao B, Wang C, Tan Y, Chen X, Liu Y, Ning L, Chen L, Li M, Liu Y, Wang G, Yuan Z, Feng Z, Zhang Y, Wu Y, Chen Y. 2020. Reduction and functional exhaustion of T cells in patients with coronavirus disease 2019 (COVID-19). Front Immunol 11:827. doi:10.3389/fimmu.2020.00827. 32425950 PMC7205903

[11] Westmeier J, Paniskaki K, Karaköse Z, Werner T, Sutter K, Dolff S, Overbeck M, Limmer A, Liu J, Zheng X, Brenner T, Berger MM, Witzke O, Trilling M, Lu M, Yang D, Babel N, Westhoff T, Dittmer U, Zelinskyy G. 2020. Impaired cytotoxic CD8^+^ T cell response in elderly COVID-19 patients. bioRxiv. https://www.biorxiv.org/content/10.1101/2020.08.21.262329v1.full.pdf. 10.1128/mBio.02243-20 PMC7502863 32948688

[12] Weiskopf D, Schmitz KS, Raadsen MP, Grifoni A, Okba NMA, Endeman H, van den Akker JPC, Molenkamp R, Koopmans MPG, van Gorp ECM, Haagmans BL, de Swart RL, Sette A, de Vries RD. 2020. Phenotype and kinetics of SARS-CoV-2-specific T cells in COVID-19 patients with acute respiratory distress syndrome. Sci Immunol 5:eabd2071. doi:10.1126/sciimmunol.abd2071. 32591408 PMC7319493

[13] Yu K. 2020. Thymosin alpha-1 protected T cells from excessive activation in severe COVID-19. Research Square rs.3.rs-25869/v1. https://www.researchsquare.com/article/rs-25869/v2.

[14] Zhang F, Gan R, Zhen Z, Hu X, Li X, Zhou F, Liu Y, Chen C, Xie S, Zhang B, Wu X, Huang Z. 2020. Adaptive immune responses to SARS-CoV-2 infection in severe versus mild individuals. Signal Transduct Target Ther 5:156. doi:10.1038/s41392-020-00263-y. 32796814 PMC7426596

[15] Dan JM, Mateus J, Kato Y, Hastie KM, Yu ED, Faliti CE, Grifoni A, Ramirez SI, Haupt S, Frazier A, Nakao C, Rayaprolu V, Rawlings SA, Peters B, Krammer F, Simon V, Saphire EO, Smith DM, Weiskopf D, Sette A, Crotty S. 2021. Immunological memory to SARS-CoV-2 assessed for up to 8 months after infection. Science 371:eabf4063. doi:10.1126/science.abf4063. 33408181 PMC7919858

[16] Chen X, Ling J, Mo P, Zhang Y, Jiang Q, Ma Z, Cao Q, Hu W, Zou S, Chen L, Yao L, Luo M, Chen T, Deng L, Liang K, Song S, Yang R, Zheng R, Gao S, Gui X, Ke H, Hou W, Lundkvist Å, Xiong Y. 2020. Restoration of leukomonocyte counts is associated with viral clearance in COVID-19 hospitalized patients. medRxiv 2020.03.03.20030437. https://www.medrxiv.org/content/medrxiv/early/2020/03/06/2020.03.03.20030437.full.pdf.

[17] Garcia-Beltran FW, Lam EC, Denis KS, Nitido AD, Garcia ZH, Hauser BM, Feldman J, Pavlovic MN, Gregory DJ, Poznansky MC, Sigal A, Schmidt AG, Iafrate AJ, Naranbhai V, Balazs AB. 2021. Multiple SARS-CoV-2 variants escape neutralization by vaccine-induced humoral immunity. Cell 184:2372–2383.e9. doi:10.1016/j.cell.2021.03.013. 33743213 PMC7953441

[18] Chandrashekar A, Liu J, Martinot AJ, McMahan K, Mercado NB, Peter L, Tostanoski LH, Yu J, Maliga Z, Nekorchuk M, Busman-Sahay K, Terry M, Wrijil LM, Ducat S, Martinez DR, Atyeo C, Fischinger S, Burke JS, Slein MD, Pessaint L, Van Ry A, Greenhouse J, Taylor T, Blade K, Cook A, Finneyfrock B, Brown R, Teow E, Velasco J, Zahn R, Wegmann F, Abbink P, Bondzie EA, Dagotto G, Gebre MS, He X, Jacob-Dolan C, Kordana N, Li Z, Lifton MA, Mahrokhian SH, Maxfield LF, Nityanandam R, Nkolola JP, Schmidt AG, Miller AD, Baric RS, Alter G, Sorger PK, Estes JD, et al. 2020. SARS-CoV-2 infection protects against rechallenge in rhesus macaques. Science 369:812–817. doi:10.1126/science.abc4776. 32434946 PMC7243369

[19] Deng W, Bao L, Liu J, Xiao C, Liu J, Xue J, Lv Q, Qi F, Gao H, Yu P, Xu Y, Qu Y, Li F, Xiang Z, Yu H, Gong S, Liu M, Wang G, Wang S, Song Z, Liu Y, Zhao W, Han Y, Zhao L, Liu X, Wei Q, Qin C. 2020. Primary exposure to SARS-CoV-2 protects against reinfection in rhesus macaques. Science 369:818–823. doi:10.1126/science.abc5343. 32616673 PMC7402625

[20] Qin C, Zhou L, Hu Z, Zhang S, Yang S, Tao Y, Xie C, Ma K, Shang K, Wang W, Tian D. 2020. Dysregulation of immune response in patients with coronavirus 2019 (COVID-19) in Wuhan, China. Clin Infect Dis 71:762–768. doi:10.1093/cid/ciaa248. 32161940 PMC7108125

[21] Huang I, Pranata R. 2020. Lymphopenia in severe coronavirus disease-2019 (COVID-19): systematic review and meta-analysis. J Intensive Care 8:36. doi:10.1186/s40560-020-00453-4. 32483488 PMC7245646

[22] Wang D, Hu B, Hu C, Zhu F, Liu X, Zhang J, Wang B, Xiang H, Cheng Z, Xiong Y, Zhao Y, Li Y, Wang X, Peng Z. 2020. Clinical characteristics of 138 hospitalized patients with 2019 novel coronavirus-infected pneumonia in Wuhan, China. JAMA 323:1061–1069. doi:10.1001/jama.2020.1585. 32031570 PMC7042881

[23] Tan L, Wang Q, Zhang D, Ding J, Huang Q, Tang Y-Q, Wang Q, Miao H. 2020. Lymphopenia predicts disease severity of COVID-19: a descriptive and predictive study. Signal Transduct Target Ther 5:33. doi:10.1038/s41392-020-0148-4. 32296069 PMC7100419

[24] Yang X, Yu Y, Xu J, Shu H, Xia J, Liu H, Wu Y, Zhang L, Yu Z, Fang M, Yu T, Wang Y, Pan S, Zou X, Yuan S, Shang Y. 2020. Clinical course and outcomes of critically ill patients with SARS-CoV-2 pneumonia in Wuhan, China: a single-centered, retrospective, observational study. Lancet Respir Med 8:475–481. doi:10.1016/S2213-2600(20)30079-5. 32105632 PMC7102538

[25] Ganji A, Farahani I, Khansarinejad B, Ghazavi A, Mosayebi G. 2020. Increased expression of CD8 marker on T-cells in COVID-19 patients. Blood Cells Molecules Dis 83:102437. doi:10.1016/j.bcmd.2020.102437. PMC7194879 32325421

[26] Shaw AC, Goldstein DR, Montgomery RR. 2013. Age-dependent dysregulation of innate immunity. Nat Rev Immunol 13:875–887. doi:10.1038/nri3547. 24157572 PMC4096436

[27] Lassaunière R, Frische A, Harboe ZB, Nielsen ACY, Fomsgaard A, Krogfelt A, Jørgensen CS. 2020. Evaluation of nine commercial SARS-CoV-2 immunoassays. medRxiv. https://www.medrxiv.org/content/10.1101/2020.04.09.20056325v1.full.pdf.

[28] Xiang J, Yan M, Li H, Liu T, Lin C, Huang S, Shen C. 2020. Evaluation of enzyme-linked immunoassay and colloidal gold-immunochromatographic assay kit for detection of novel coronavirus (SARS-Cov-2) causing an outbreak of pneumonia (COVID-19). medRxiv. https://www.medrxiv.org/content/10.1101/2020.02.27.20028787v1.

[29] Zhao J, Zhao J, Perlman S. 2010. T cell responses are required for protection from clinical disease and for virus clearance in severe acute respiratory syndrome coronavirus-infected mice. J Virol 84:9318–9325. doi:10.1128/JVI.01049-10. 20610717 PMC2937604

[30] Chen J, Lau YF, Lamirande EW, Paddock CD, Bartlett JH, Zaki SR, Subbarao K. 2010. Cellular immune responses to severe acute respiratory syndrome coronavirus (SARS-CoV) infection in senescent BALB/c mice: CD4 T cells are important in control of SARS-CoV infection. J Virol 84:1289–1301. doi:10.1128/JVI.01281-09. 19906920 PMC2812346

[31] Swain SL, McKinstry KK, Strutt TM. 2012. Expanding roles for CD4^+^ T cells in immunity to viruses. Nat Rev Immunol 12:136–148. doi:10.1038/nri3152. 22266691 PMC3764486

[32] Elong Ngono A, Young MP, Bunz M, Xu Z, Hattakam S, Vizcarra E, Regla-Nava JA, Tang WW, Yamabhai M, Wen J, Shresta S. 2019. CD4^+^ T cells promote humoral immunity and viral control during Zika virus infection. PLoS Pathog 15:e1007474. doi:10.1371/journal.ppat.1007474. 30677097 PMC6345435

[33] Braun J, Loyal L, Frentsch M, Wendisch D, Georg P, Kurth F, Hippenstiel S, Dingeldey M, Kruse B, Fauchere F, Baysal E, Mangold M, Henze L, Lauster R, Mall MA, Beyer K, Röhmel J, Schmitz J, Miltenyi S, Muller MA, Witzenrath M, Suttorp N, Kern F, Reimer U, Wenschuh H, Drosten C, Corman VM, Giesecke-Thiel C, Sander LE, Thiel A. 2020. Presence of SARS-CoV-2-reactive T cells in COVID-19 patients and healthy donors. medRxiv. https://www.medrxiv.org/content/10.1101/2020.04.17.20061440v1. 10.1038/s41586-020-2598-9 32726801

[34] Chen G, Wu D, Guo W, Cao Y, Huang D, Wang H, Wang T, Zhang X, Chen H, Yu H, Zhang X, Zhang M, Wu S, Song J, Chen T, Han M, Li S, Luo X, Zhao J, Ning Q. 2020b. Clinical and immunological features of severe and moderate coronavirus disease 2019. J Clin Invest 130:2620–2629. doi:10.1172/JCI137244. 32217835 PMC7190990

[35] Calvet J, Gratacós J, Amengual MJ, Llop M, Navarro M, Moreno A, Berenguer-Llergo A, Serrano A, Orellana C, Cervantes M. 2020. CD4 and CD8 lymphocyte counts as surrogate early markers for progression in SARS-CoV-2 pneumonia: a prospective study. Viruses 12:1277. doi:10.3390/v12111277. 33182268 PMC7695272

[36] Jin M, Shi N, Wang M, Shi C, Lu S, Chang Q, Sha S, Lin Y, Chen Y, Zhou H, Liang K, Huang X, Shi Y, Huang G. 2020. CD45: a critical regulator in immune cells to predict severe and non-severe COVID-19 patients. Aging (Albany NY) 12:19867–19879. doi:10.18632/aging.103941. 33065551 PMC7655207

[37] Panda S, Ding JL. 2015. Natural antibodies bridge innate and adaptive immunity. J Immunol 194:13–20. doi:10.4049/jimmunol.1400844. 25527792

[38] Chowdhury MA, Hossain N, Kashem MA, Shahid MA, Alam A. 2020. Immune response in COVID-19: a review. J Infect Public Health 13:1619–1629. doi:10.1016/j.jiph.2020.07.001. 32718895 PMC7359800

[39] Long Q, Liu B, Deng H, Wu G, Deng K, Chen Y, Liao P, Qiu J, Lin Y, Cai X, Wang D, Hu Y, Ren J, Tang N, Xu Y, Yu L, Mo Z, Gong F, Zhang Z, Tian W, Hu L, Zhang X, Xiang J, Du H, Liu H, Lang C, Luo X, Wu S, Cui X, Zhou Z, Zhu M, Wang J, Xue C, Li X, Wang L, Li Z, Wang K, Niu C, Yang Q, Tang X, Zhang Y, Liu X, Li J, Zhang D, Zhang F, Liu P, Yuan J, Li Q, Hu J, Chen J, et al. 2020. Antibody responses to SARS-CoV-2 in patients with COVID-19. Nat Med 26:845–848. doi:10.1038/s41591-020-0897-1. 32350462

[40] Hou H, Wang T, Zhang B, Luo Y, Mao L, Wang F, Wu S, Sun Z. 2020. Detection of IgM and IgG antibodies in patients with coronavirus disease 2019. Clin Transl Immunol 9:e01136. 10.1002/cti2.1136 PMC7202656 32382418

[41] Liu X, Wang J, Xu X, Liao G, Chen Y, Hu C. 2020. Patterns of IgG and IgM antibody response in COVID-19 patients. Emerg Microbes Infect 9:1269–1274. doi:10.1080/22221751.2020.1773324. 32515684 PMC7448841

[42] Li G, Chen X, Xu A. 2003. Profile of specific antibodies to the SARS-associated coronavirus. N Engl J Med 349:508–509. doi:10.1056/NEJM200307313490520. 12890855

[43] Sun B, Feng Y, Mo X, Zheng P, Wang Q, Li P, Peng P, Liu X, Chen Z, Huang H, Zhang F, Luo W, Niu X, Hu P, Wang L, Peng H, Huang Z, Feng L, Li F, Zhang F, Li F, Zhong N, Chen L. 2020. Kinetics of SARS-CoV-2 specific IgM and IgG responses in COVID-19 patients. Emerg Microbes Infect 9:940–948. doi:10.1080/22221751.2020.1762515. 32357808 PMC7273175

