# Supplementary material for: Evaluation of the Levels of Peripheral CD3+, CD4+, and CD8+ T Cells and IgG and IgM Antibodies in COVID-19 Patients at Different Stages of Infection
Source: Microbiol Spectr. 2022 Feb 23;10(1):e00845-21. doi: 10.1128/spectrum.00845-21 (PMC8865559; doi:10.1128/spectrum.00845-21)
Supplement: SUPPLEMENTAL FILE 1 — Supplemental material. Download SPECTRUM00845-21_Supp_1_seq4.pdf, PDF file, 0.2 MB [file spectrum00845-21_supp_1_seq4.pdf]

Supplementary Section:

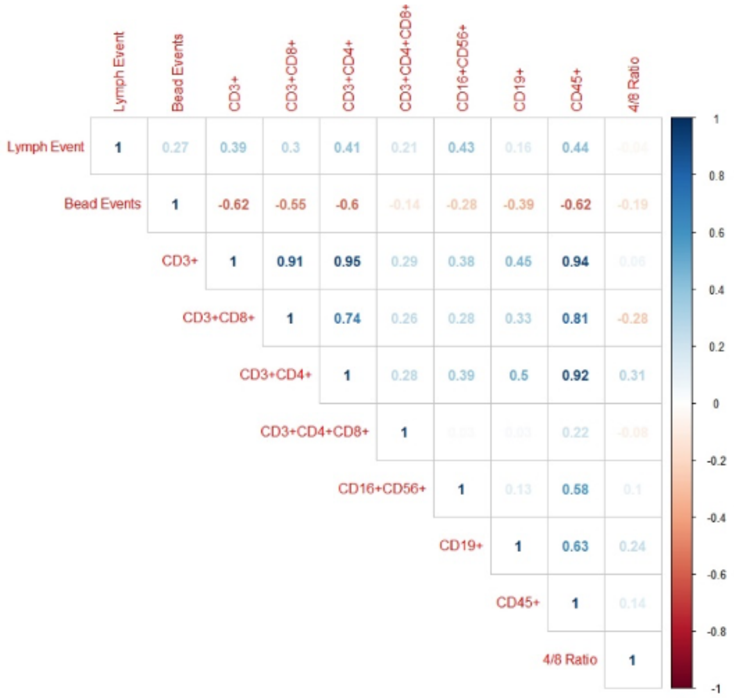

Fig. S1: Correlation Matrix Plot. The plot presents the multicollinearity between the variables (lymph event, bead events, CD3+, CD3+ CD8+, CD3+ CD4+, CD3+ CD4+ CD8+, CD16+ CD56+, CD19+, CD45+, 4/8 Ratio) and their behaviour.
